# Retention in Georgia opioid substitution therapy program and associated factors

**DOI:** 10.1186/s12954-016-0124-z

**Published:** 2016-12-08

**Authors:** Ekaterine Ruadze, Khatuna Todadze

**Affiliations:** 1 The National Center for Disease Control and Public Health, 9 Asatiani Street, Tbilisi, Georgia; 2 Center for Mental Health and Prevention of Addiction, 21 Kavtaradze Street, Tbilisi, Georgia; 3 Addiction Department, Tbilisi State Medical University, 7 Asatiani Street, Tbilisi, Georgia

**Keywords:** Substance abuse, Retention rate, Opioid substitute treatment, Detoxification

## Abstract

**Background:**

Substance abuse has been considered as a growing challenge in Georgia that is closely linked with human immune deficiency virus (HIV) and hepatitis C transmission due to unsafe injection and other uncontrolled behaviors. Methadone maintenance therapy is one of the major treatment options for opioid-dependent individuals. It has proven efficacy in decreasing illegal opioid consumption and criminal behavior as well as reducing the level of HIV infection, mortality, HCV infection, and increasing social functioning.

**Methods:**

The data was initially extracted from the electronic database, as of October 30, 2015, for the patients undergoing methadone maintenance therapy in 2014 and 2015. We used two types of statistical analysis: binary regression and time-to-event analysis (Kaplan-Meier). For binary regression analysis, patients who initiated the treatment 12, 9, 6, and 3 months prior to October 30, 2015, respectively, were eligible for >12-, >9-, >6-, and >3-month retention analysis. We identified two types of the retention periods: (I) “the program specific retention period” (the time spent (uninterruptedly) in the Global Fund to Fight HIV/AIDS, Tuberculosis, and Malaria (GFATM) opioid substitution treatment (OST) program after the clients’ last entry) and (II) “being on OST retention period” (the time spent (uninterruptedly) on OST since the clients’ last entry).

For time-to-event analysis, the two different endpoints were investigated: (i) dropouts and (ii) being detained.

**Results:**

The analysis showed that at each time point, “being on OST retention” rates are slightly higher than “program specific retention” rates. The percentages of patients retained in OST treatment after 3, 6, 9, and 12 months from the initiation of the treatment, respectively, were 89, 86, 85, and 83% and the percentages of patients retained in the GFATM program at the same time points were 88, 83, 82, and 80%. Patients older than 40 years are twice as likely to stay in the program compared to younger individuals. Gender is only associated with >9 and >12 months retention with approximately three times the odds for men compared to women. The strength of the association between hepatitis C status and “program specific” retention increases with time spent in the program as *p* values decrease from 0.07 for >3- and >6-month retention to 0.01 for >9- and >12-month retention. The younger age group was more likely to get dropouts and be detained. HIV status and social status did not show statistically significant association with retention.

**Conclusions:**

These findings identify the need for more support for younger patients as they are more vulnerable to dropouts and detention compared to the older age group, especially during the early stage of treatment.

## Background

Substance abuse has been considered a growing challenge in Georgia that is closely linked with human immune deficiency virus (HIV) and hepatitis C transmission due to unsafe injection and other uncontrolled behaviors. According to studies conducted in 2009, 2012, and 2015, the number of people who inject drugs has increased from 40,000 to 45,000 and then to 49,700 [1–3].

The estimated HIV prevalence in Georgia is 0.3% (0.2–0.4%) among the adult population (15–49 years of age). Injecting drug use was considered the leading route of HIV transmission in the early stages of the HIV epidemic in Georgia. Since 2012, however, heterosexual intercourse has become the major route of transmission (44% in 2012, 49% in 2013, and 45% in 2014) [4]. According to the Infectious Disease, AIDS and Clinical Immunology Center, HIV infection acquired through injecting drug use accounts for 45.9% of cases [5].

Worldwide, there are 12 million people who inject drugs (PWID), of whom 14.0% are living with HIV [6].

The comparative analysis of Bio-Behavioral Surveillance Survey (BBS) results conducted in the last 5 years (2009–2014) showed significant changes in the injecting drug scene [2, 7, 8]. Heroin is the most commonly misused substance among drug users in Georgia. Use of heroin dropped in 2012 by 23.3% compared to 2009 and increased again in 2015 reaching 58.1%. Changes in buprenorphine (the second most frequently injected drug) misuse had a similar pattern to heroin; it dropped from 43.3% in 2009 to 13.4% and then increased in 2015 to 25.9%. Self-made desomorphine, containing the substance known as “Krokodile,” was first captured by BBS studies in 2012. This drug is a cheaper alternative to heroin and shows high potential to cause dependence and injecting-related harm. However, in 2015, self-made injecting drug use, including desomorphine, was reported by a lower proportion of PWIDs compared to 2012.

In 2014, the opiates affected some 17 million people in the world. The decline of opium production in 2015 is unlikely to lead to shortages in the global heroin market given the high opium production levels of previous years [6].

Methadone maintenance therapy is one of the major treatment options for opioid-dependent individuals. It has proven efficacy in decreasing illegal opioid consumption and criminal behavior as well as reducing the level of HIV infection, mortality, hepatitis C virus (HCV) infection, and social functioning. There are studies reporting a positive impact of retention in opioid substitution treatment (OST) on HIV outcomes: the longer the duration of retention in OST, the higher the likelihood of long-term virological success [9, 10].

OST was introduced in Georgia in 2005. Nowadays, OST is functional through three sources: (I) the donor: The Global Fund to Fight HIV/AIDS, Tuberculosis, and Malaria (GFATM), (II) the state program, and (III) the private sector. There are two different OST programs available: (1) methadone maintenance program and (2) the program using combined preparation with buprenorphine and naloxone.

The state program is based on the co-payment principle: the cost of the methadone is covered by the State, while services (visit to doctor, counseling, urine-testing, etc.) are self-paid (out-of-pocket) by patients at GEL 110 a month. The co-payment does not apply to HIV-positive individuals and those under the poverty line. In total, there are 15 sites operated by the State throughout the country. In 2015, the country’s total capacity for OST was 2750 (the state program had 2000 patients and the GFATM 750 patients) patients.

**Fig. 1:**
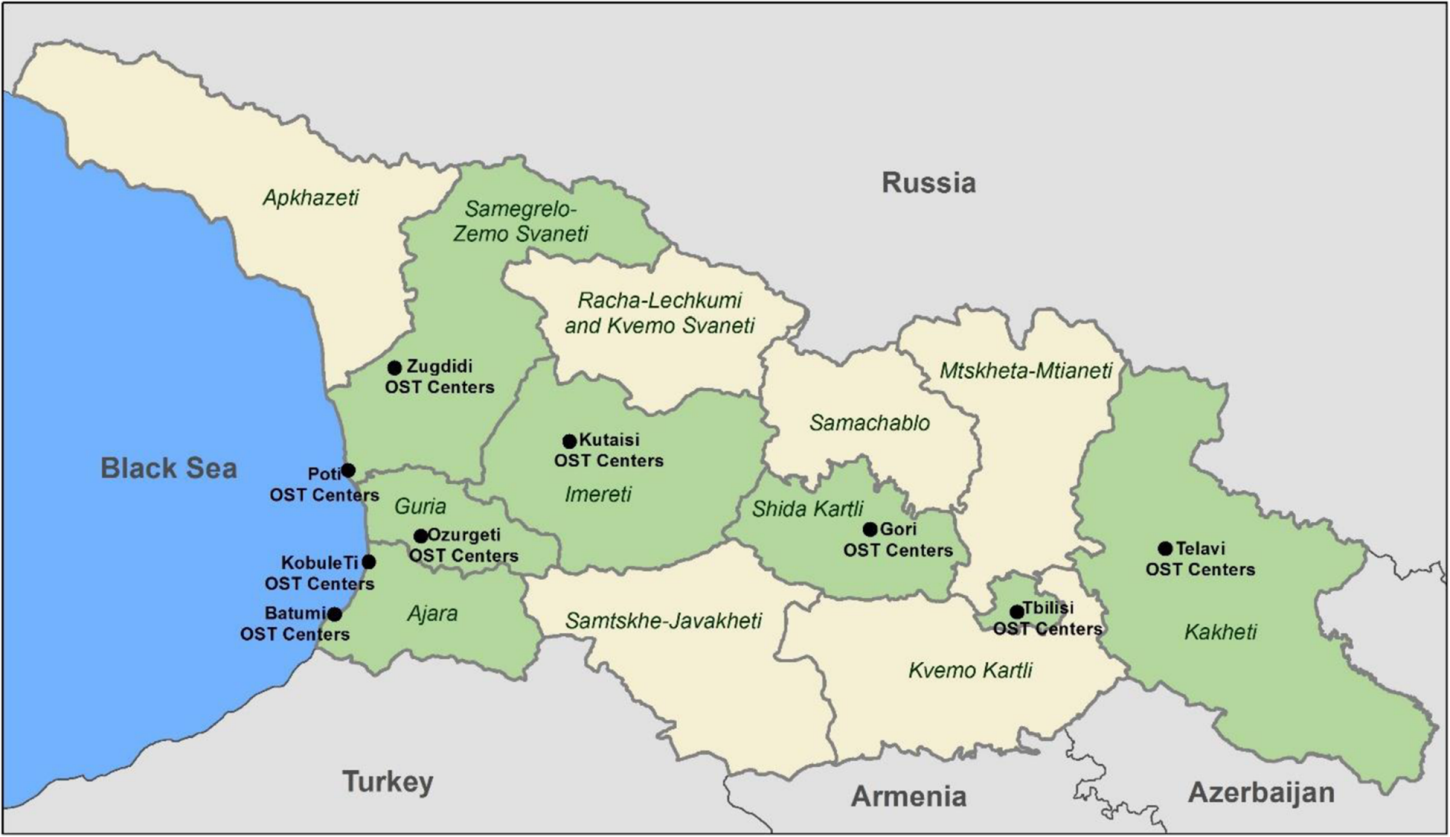
OST coverage—Georgia;  Regions with OST centers;  Other regions

The National Center for Disease Control and Public Health (NCDC) of Georgia through the GFATM-funded HIV prevention program provides treatment at six OST sites. There are four sites in Tbilisi (the capital city), one in Gori (east Georgia), and one in Batumi (southwest Georgia). Additionally, two sites are running in the penitentiary institutions—one in Tbilisi and another in Kutaisi (west Georgia), providing long-term detoxification of methadone. Figure 1 represents the country’s OST total coverage.

We conducted the operational study to investigate retention in OST and associated factors.

## Methods

GFATM OST program uses an electronic web-based real-time data collection tool in which the data is entered on a daily basis.

The clients’ information which are routinely collected are (1) client’s ID, (2) residency address, (3) date of birth, (4) gender, (5) date of program entry, (6) date of program cessation, (7) reason of cessation, (8) OST program provider name and address, (9) hepatitis C status, (10) acquired immune deficiency syndrome (AIDS) status, and (11) social status.

The data was initially extracted from the electronic database as of October 30, 2015, for the patients undergoing methadone maintenance therapy in 2014 and 2015. All duplicates as well as observations for which the program entry date was missing were excluded. The final data set included 1051 individuals out of an initial 1249.

### Definitions

The database consisted of patients (i) transferred from other OST programs into the GFATM OST program and (ii) those originally entered in the GFATM OST program. For this reason, we identified two types of the retention periods: (I) “the program specific retention period” and (II) “being on OST retention period.”

1. “The program specific retention period” was defined as the time spent (uninterruptedly) in the GFATM OST program after the clients’ last entry.
2. “Being on OST retention period” was defined as the time spent (uninterruptedly) in OST since the clients’ last entry.
  For those patients which only had GF OST program experience “the program specific retention period” and “being on OST retention period” coincided with each other.
3. “Dropout” was defined as a failure to adhere to the regimen (e.g., frequent use of additional narcotics or alcohol, missed sessions more than ten times, incorrect code of conduct towards the staff, attempt of drug stealing or drug dealing at the service area) or abandoning the program with or without notification.

### Binary regression analysis

**Fig. 2:**
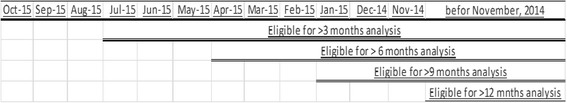
Clients’ eligibility chart for >3, >6, >9, and >12-month retention analysis

Patients who initiated the treatment 12, 9, 6, and 3 months prior to October 30 were, respectively, eligible for >12-, >9-, >6-, and >3-month retention analysis (Fig. 2).

**Fig. 3:**
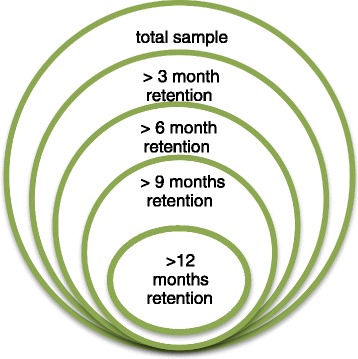
The sample composition for each time point

The outcome variable (retention) at different time points was calculated as follows: (I) more than 3-month retention ≥90 days stay, (II) more than 6-month retention ≥181 days stay, (III) more than 9-month retention ≥270 days stay, and (IV) more than 12-month retention ≥365 days stay (Fig. 3).

The independent variables were age at program entry (grouped at median value ≤40 and >40 years old and as a continuous variable), hepatitis C status, HIV status, gender, and being socially vulnerable. The latter was defined as a patient who applied to the Social Service Agency (SSA) in order to receive social assistance. The database automatically captures the information about the obtained social scores of the patient (at the moment of the patient’s registration), if this patient has applied for the social assistance and therefore is registered into the SSA database.

First, univariate analyses were performed to assess the strength of the association between each independent variable and the outcome. Other variables were then included in an initial multivariable model if they had a univariate *p* value of <0.05 and showed statistically significant association with the outcome as well as with other exposure variables. In the final model, variables were selected based on a significance threshold of *p* < 0.05. Separate multivariable modes were built for “program specific” and “OST specific” retention periods.

Results are presented as estimated odds ratios (OR) with corresponding 95% confidence intervals (CI) and *p* values. All *p* values are two-sided and reported to three decimal places with those less than 0.001 reported as *p* < 0.001.

### Time-to-event analysis

Factors that showed statistically significant association with retention in OST were further studied in relation to two different endpoints (failure events): (I) dropouts and (II) being detained, by contracting Kaplan-Meier survival curves. Patient’s death or retention in the program until the end of the study period as well as residential address or clinic change was treated as censored data.

Due to the huge inequality in gender distribution within the cohort (29 females vs. 1022 males), we did not put gender as an exposure variable for time-to-event analysis.

Analyses were performed using STATA version 11.

## Results

### Retention

Table 1 represents characteristics of all individuals in the data set. Age ranged from 21 to 67 years with the mean age of 40.4 years. Of the cohort, 97.2% were male, 4.19% were HIV-positive, 48.8% were hepatitis C virus-positive, 21.5% were socially vulnerable, and 27.7% transferred into the GFATM OST program from other OST programs.

**Table 1:** Characteristics of the cohort (*n* = 1051)

	% of patients	*n*/*N* of patients	Median; IQR [25–75%]
Age			40 [34–46]
Age group 1 (≤40)	49%	515/1051	
Age group 2 (>40)	51%	536/1051	
Gender			
Male	97.2%	1022/1051	
Female	2.8%	29/1051	
Socially vulnerable	21.5%	226/1051	
Hepatitis C status (confirmed cases)	48.8%	513/1051	
HIV status	4.19%	44/1051	
Came from other OST program/transfers	27.7%	291/1051	
Retention in GF program (months)			8.0; IQR [2.9–31.5]
Retention in OST (months)			13.3; IQR [4.2–37.7]
>3-month retention	80.3%	844/1051	
>6-month retention	67.5%	710/1051	
>9-month retention	60.1%	632/1051	
>12-month retention	52.9%	556/1051	

The analysis showed that at each time point, “being on OST retention” rates are slightly higher than “program specific retention” rates. The percentages of patients retained in OST treatment after 3 months, 6, 9, and 12 months from the initiation of the treatment, respectively, were 89, 86, 85, and 83% and the percentages of patients retained in the GFATM program at the same time points were 88, 83, 82, and 80%.

In univariate analysis, age showed a strong statistical association with both “program specific” and “being on OST specific” retention periods. Generally, at each time point, patients older than 40 years have almost two times the odds of staying in the program compared to younger individuals. Per each year increase in age, the odds of retention increases ~7%. Gender was associated with only >9- and >12-month retention with about three times the odds for men compared to women. Positive hepatitis C status was only associated with “program specific” retention periods at >9-month (1.73 95% CI (1.15–2.63) and >12-month (1.76 95% CI (1.15–2.67) time points. HIV status and social status did not show a statistically significant association with retention (Table 2).

**Table 2:** Results of univariate analysis of association between the exposure variables and retention at different time points (*the first row represents the results for program specific data and the second row for “being on OST” data*)

	>3-month retention		>6-month retention	
	OR (95% CI)	*p* value	OR (95% CI)	*p* value
Age (>40 vs. ≤40 years)	2.47 (1.62–3.77)	*<0.001*	1.89 (1.27–2.81)	*0.002*
	2.38 (1.54–3.67)	*<0.001*	2.04 (1.37–3.04)	*<0.001*
Age (cont. variable)	*1.07 (1.04–1.10)*	*<0.001*	*1.06 (1.03–1.08)*	*<0.001*
	*1.07 (1.04–1.10)*	*<0.001*	*1.06 (1.03–1.08)*	*<0.001*
Gender (male vs. female)	0.98 (0.29–3.33)	0.974	1.57 (0.56–4.43)	0.385
	1.14 (0.34–3.89)	0.830	1.86 (0.67–5. 24)	0.224
Socially vulnerable	1.20 (0.73–1.97)	0.463	1.35 (0.83–2.21)	0.232
	1.18 (0.71–1.98)	0.519	1.21 (0.75–1.96)	0.433
Hepatitis C status	*1.45 (0.97–2.17)*	*0.071*	1.43 (0.97–2.11)	*0.072*
	1.25 (0.83–1.90)	0.281	1.02 (0.69–1.51)	0.908
HIV status	0.82 (0.31–2.18)	0.702	0.78 (0.33–1.85)	0.583
	0.73 (0.28–1.93)	0.528	0.61(0.26–1.44)	0.265
	>9-month retention		>12-month retention	
Age (>40 vs. ≤40 years)	2.23 (1.46–3.41)	*<0.001*	2.49 (1.62–3.83)	*<0.001*
	2.41(1.59–3.66)	*<0.001*	2.44 (1.59–3.73)	*<0.001*
Age (cont. variable)	*1.07 (1.04–1.10)*	*<0.001*	*1.07 (1.04–1.10)*	*<0.001*
	*1.07 (1.05–1.10)*	*<0.001*	*1.08 (1.05–1.11)*	*<0.001*
Gender	3.21 (1.28–8.07)	*0.013*	2.46 (0.95–6.41)	*0.065*
	3.69 (1.49–9.14)	*0.005*	2.89 (1.13–7.43)	*0.027*
Socially vulnerable	1.19 (0.72–1.98)	0.487	1.14 (0.69–1.86)	0.612
	1.17 (0.71–1.92)	0.534	1.18 (0.72–1.93)	0.515
Hepatitis C status	*1.73 (1.15–2.63)*	*0.009*	*1.76 (1.15–2.67)*	*0.009*
	1.18 (0.79–1.78)	0.403	1.23 (0.82–1.86)	0.318
HIV status	0.77 (0.32–1.82)	0.550	1.14 (0.42–3.07)	0.797
	0.61 (0.26–1.45)	0.265	0.89 (0.33–2.39 )	0.816

*Age (cont. variable) analysis “age” as a continious variable i.e. examains the retention per each year increase in age*

In multivariable analysis, age remained as a strong predictor for retention. After adjusting for gender and hepatitis C status, the odds of patents’ retention increased by ~6% per each year increase of age. Hepatitis C status and gender, after adjusting for age (as a group variable), showed some statistically significant association with retention (Table 3).

**Table 3:** Multivariate analysis at >9- and >12-month time points (gender and hep C status are adjusted for age as a group variable)

	Model I: program specific		Model II: being on OST	
	OR 95% CI	*P* values	ORs 95% CI	*p* values
9 months				
Age (>40 vs. ≤40 years)	2.05 (1.34–3.15)	<0.001	2.33 (1.53–3.55)	<0.001
Age (cont. variable)	1.06 (1.03–1.08)	<0.001	1.06 (1.05–1.09)	<0.001
Gender (male vs. female)	2.71 (1.06–6.95)	0.038	3.18 (1.26–8.0)	0.014
Hepatitis C status	1.55 (1.01–2.37)	0.042		
12 months				
Age (>40 vs. ≤40 years)	2.36 (1.53–3.64)	<0.001	2.37 (1.55–3.64)	<0.001
Age (cont. variable)	1.06 (1.04–1.09)	<0.001	1.06 (1.04–1.10)	<0.001
Gender (male vs. female)			2.48 (0.95–6.48)	0.064
Hepatitis C status	1.58 (1.03–2.44)	0.034		

### Cessation

From the total cohort, 32% (341) left the program, 2.3% died (10), 14.1% (48) was detained, 19% (65) left the program after the detoxification (in this case, this is the planned gradual reduction of methadone doses), 35% (120) ceased the treatment voluntarily (with or without any notification or due to failure to keep the regimen), 16.4% (56) changed to another clinic, and 12.3% (42) changed the residential address. For each reason of cessation, younger age group patients (<40) were proportionally more compared to older age group patients, at *p* = 0.014 significant level.

For the patients who left the OST program, the median duration of stay in the OST program was 140 days (interquartile (IQR) 31–558). The longest median duration of stay (335 days IQR, 122–745 days]) was observed among patients who left the program after the detoxification course, followed by detainees with 140 days (IQR, 60–574) and dropouts with 56 days (IQR, 23–190).

**Fig. 4:**
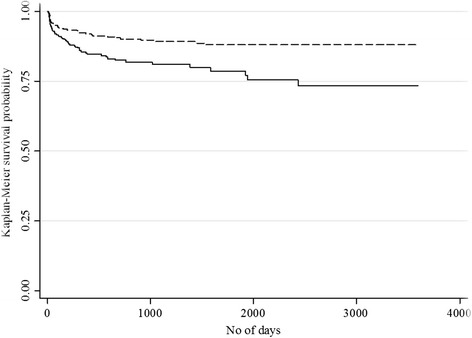
Kaplan-Meier survival curve for length of time since inclusion in OST until occurrence of dropouts;  age ≤40; age >40; *p* < 0.001

**Fig. 5:**
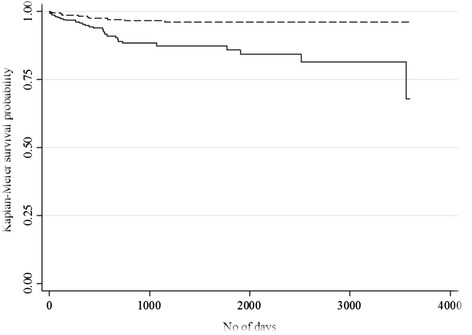
Kaplan-Meier survival curve for length of time since inclusion in OST until occurrence of detention;  age ≤40; age >40; *p* < 0.001

There was a significant difference in dropout and detention times between the age groups (log rank tests for both outcomes: *p* < 0.001). For the younger age group patients, the probability of experiencing the dropouts some time after the end of the observation period was about 0.73 (95% CI 0.66–0.81), and for the old age group, it was 0.88 (95% CI 0.84–0.91). The younger age group was also less likely to be detained sometime after the observation period compared to the old age group (0.67 (95% CI 0.36–0.86) vs. 0.95 (95% CI 0.93–0.98)) (Figs. 4 and 5).

**Fig. 6:**
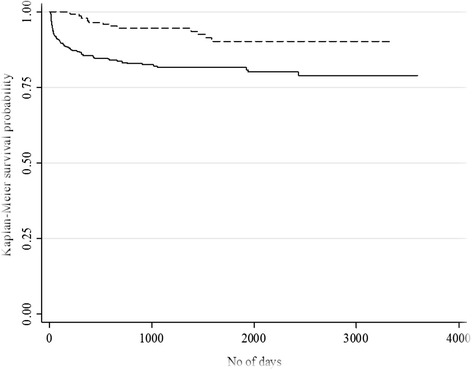
Kaplan-Meier survival curve for length of time since inclusion in OST until occurrence of dropouts;  not transfers; transfers; *p* < 0.001

Kaplan-Meier dropout probability estimates at the end of the observation period were about 0.90 (95% CI 0.83–0.94) for the transferred patients and 0.79 (95% CI 0.74–0.83) for not transferred patients (log rank test *p* < 0.001). There was no statistically significant difference in Kaplan-Meier detention probability estimates (0.80(95% CI 0.53–0.92) vs. 0.92 (95% CI (0.78–0.96), transferred vs. not transferred, log rank test *p* = 0.059) (Fig. 6).

The occurrences of the endpoints between the groups of hepatitis C-positive and -negative patients were not statistically significant (log rank test for dropouts *p* = 0.705 and for detention *p* = 0.148).

## Discussion

### Key results

Our analysis showed that age is a strong predictor for retention. The odds of retention in OST enhance per each year increase of age by 6%. In addition, patients over 40 years of age retain longer and are less likely to dropout or to be detained. Positive hepatitis C status is associated with relatively longer retention periods (>9 and >12 months). Patients who came from the paid program to the free-of-charge program have a higher probability of experiencing dropouts or being detained at some point after the end of the observation period.

### Strengths and limitations

The strength of the study is that the data was extracted from a real-time web-based electronic system which captures and registers the basic characteristics of the patients. The accuracy of the data is monitored on a quarterly basis.

The results of association between gender and retention were not conclusive as females represent only 2.8% of the cohort (29/1051).

### Comparability with other studies

Due to different settings and definitions used in other studies, the comparability of our findings is also limited. Our study presents relatively high retention rates on OST >80% even after 12 months of treatment, compared to other studies’ findings. In a Ukrainian study, retention rate was 67% at 12 months of treatment [11]. Our findings are also highly compared to the WHO collaborative study that included low-, middle-, and high-income countries (averaging approximately 70% at 6 months) [12]. Hans-Ulrich Wittchen et al. found that the retention rate in Germany at 12 months was 75% [13]. Six-month treatment retention rates observed in our study were similar to those found by Ukrainian researchers [14, 15]. Such differences could be explained by the different methodology used in different studies. When we did a cross-sectional analysis of the retention rates our findings were closer to what was observed in other studies (Table 1) [11–13]. However, after excluding those not eligible for the >3-, >6-, >9-, and >12-month retention, the rates become high.

Age, gender, and hepatitis C status were not associated with﻿ retention at 6 or 12 months in a Ukrainian study [11]. Soyka M. et al. found that younger age associates with premature dropouts from the OST [16].

Being transferred from the paid program to the free-of-charge program shows a positive association with retention. These findings are consistent with Australian study findings, conducted in 2013, which suggest that dispensing fees have a negative impact on OST retention as well as lifestyle and treatment. They also suggest that sponsorship “… would potentially increase the retention rate of income-poor OST program participants” [17].

In Georgia, individuals can apply for the social benefit at the SSA. The authorized person of the SSA (the social agent) visits the family. During the visit, a “family declaration” is filled in about the social-economic state of the family on the basis of the information delivered by the authorized person of the family. After allocation of the information provided in the declaration in the unified database and processing by established methodology, a rating score is assigned to the family, which amount defines the right of the family on any benefit (pecuniary social assistance, medical insurance, etc.) [18]. Individuals who score below 70,000 are eligible for free-of-charge state OST program [19]. However, for this analysis, all individuals who applied for social status were classified as socially vulnerable regardless of their obtained scores. We took this approach since everyone who applies for the social benefit considers themselves to be poor enough to be eligible for the support. In our cohort, the median score obtained from the SSA was 77,610 [IQR, 45,330–115,750] and mean value was 93,402.

Being socially vulnerable does not appear to be the driving force for transferring; as in our cohort, socially vulnerable people are equally distributed among those transferred and not transferred from the paid OST program. Among those who came from the other OST program, 77% were not socially vulnerable. Their transfer might indicate that, despite their social status, people find it difficult to pay the OST fee. Our findings might also suggest that being transferred from the paid program to the unpaid program could be a motivator for better retention rates. As it is noted in the World Drug Report, 2016: “higher social-economic groups have a greater propensity to initiate drug use than lower social-economic groups, but it is the lower social-economic groups that pay the higher price as they are more likely to become drug dependents” [6].

## Conclusions

These findings identify the need for more support for younger patients as they are more vulnerable to dropouts and detention compared to the older age group, especially at the early stage of treatment. More analysis enabling a direct comparison between paid and free-of-charge programs is needed to assess the retention and associated factors.

## Abbreviations

AIDS: Acquired immune deficiency syndrome
BBS: Bio-Behavioral Surveillance
GFATM: Global Fund to Fight HIV/AIDS, Tuberculosis, and Malaria
HCV: Hepatitis C virus
HIV: Human immune deficiency virus
NCDC: National Center for Disease Control and Public Health
OST: Opioid substitution treatment
PWID: People who inject drugs
SSA: Social Service Agency
